# 2-(5-Fluoro-7-methyl-3-methyl­sulfanyl-1-benzofuran-2-yl)acetic acid

**DOI:** 10.1107/S1600536811026729

**Published:** 2011-07-09

**Authors:** Pil Ja Seo, Hong Dae Choi, Byeng Wha Son, Uk Lee

**Affiliations:** a Department of Chemistry, Dongeui University, San 24 Kaya-dong Busanjin-gu, Busan 614-714, Republic of Korea; b Department of Chemistry, Pukyong National University, 599-1 Daeyeon 3-dong, Nam-gu, Busan 608-737, Republic of Korea

## Abstract

The title compound, C_12_H_11_FO_3_S, was prepared by alkaline hydrolysis of ethyl 2-(5-fluoro-7-methyl-3-methyl­sulfanyl-1-benzofuran-2-yl)acetate. In the crystal, the carboxyl groups are involved in inter­molecular O—H⋯O hydrogen bonds, which link the mol­ecules into centrosymmetric dimers.

## Related literature

For the pharmacological activity of benzofuran compounds, see: Aslam *et al.* (2009 ▶); Galal *et al.* (2009 ▶); Khan *et al.* (2005 ▶). For natural products with benzofuran rings, see: Akgul & Anil (2003 ▶); Soekamto *et al.* (2003 ▶). For structural studies of related 2-(5-halo-3-methyl­sulfanyl-1-benzofuran-2-yl)acetic acid derivatives, see: Choi *et al.* (2009**a* ▶,b* ▶).
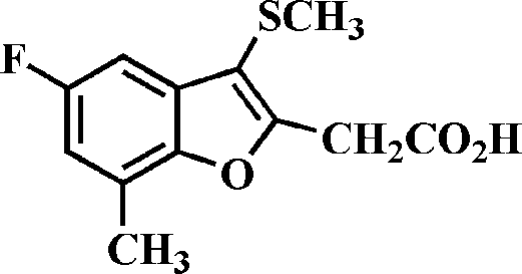


## Experimental

### 

#### Crystal data

C_12_H_11_FO_3_S
*M* *_r_* = 254.27
Monoclinic, 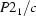

*a* = 16.1747 (9) Å
*b* = 4.9242 (3) Å
*c* = 14.4572 (7) Å
β = 91.701 (3)°
*V* = 1150.97 (11) Å^3^
*Z* = 4
Mo *K*α radiation
μ = 0.29 mm^−1^
*T* = 173 K
0.42 × 0.19 × 0.09 mm

#### Data collection

Bruker SMART APEXII CCD diffractometer
Absorption correction: multi-scan (*SADABS*; Bruker, 2009 ▶) *T*_min_ = 0.889, *T*_max_ = 0.975
10241 measured reflections
2873 independent reflections
2114 reflections with *I* > 2σ(*I*)
*R*_int_ = 0.050

#### Refinement

*R*[*F* ^2^ > 2σ(*F* ^2^)] = 0.048
*wR*(*F* ^2^) = 0.153
*S* = 1.05
2873 reflections
156 parameters
H-atom parameters constrained
Δρ_max_ = 0.38 e Å^−3^
Δρ_min_ = −0.27 e Å^−3^

### 

Data collection: *APEX2* (Bruker, 2009 ▶); cell refinement: *SAINT* (Bruker, 2009 ▶); data reduction: *SAINT*; program(s) used to solve structure: *SHELXS97* (Sheldrick, 2008 ▶); program(s) used to refine structure: *SHELXL97* (Sheldrick, 2008 ▶); molecular graphics: *ORTEP-3* (Farrugia, 1997 ▶) and *DIAMOND* (Brandenburg, 1998 ▶); software used to prepare material for publication: *SHELXL97*.

## Supplementary Material

Crystal structure: contains datablock(s) global, I. DOI: 10.1107/S1600536811026729/xu5266sup1.cif

Structure factors: contains datablock(s) I. DOI: 10.1107/S1600536811026729/xu5266Isup2.hkl

Supplementary material file. DOI: 10.1107/S1600536811026729/xu5266Isup3.cml

Additional supplementary materials:  crystallographic information; 3D view; checkCIF report

## Figures and Tables

**Table 1:** Hydrogen-bond geometry (Å, °)

*D*—H⋯*A*	*D*—H	H⋯*A*	*D*⋯*A*	*D*—H⋯*A*
O2—H2⋯O3^i^	0.84	1.87	2.706 (2)	177

Symmetry code: (i) 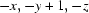.

**Fig. 1.**
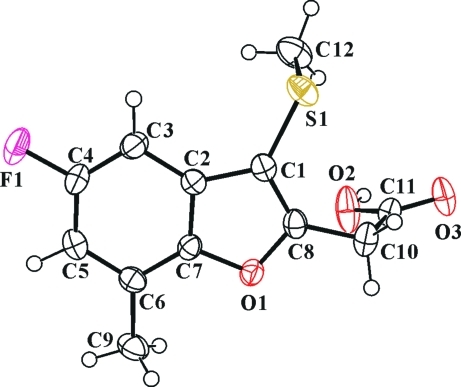
The molecular structure of the title compound with the atom numbering scheme. Displacement ellipsoids are drawn at the 50% probability level. H atoms are presented as a small spheres of arbitrary radius.

## supplementary crystallographic information

### Comment

Recently, many compounds involving a benzofuran moiety have drawn much attention due to their valuable pharmacological properties such as antibacterial and antifungal, antitumor and antiviral, and antimicrobial activities (Aslam *et al.*, 2009, Galal *et al.*, 2009, Khan *et al.*, 2005). These benzofuran derivatives occur in a wide range of natural products (Akgul & Anil, 2003; Soekamto *et al.*, 2003). As a part of our ongoing study of the substituent effect on the solid state structures of 2-(5-halo-3-methylsulfanyl-1-benzofuran-2-yl) acetic acid analogues (Choi *et al.*, 2009**a*,b*), we report herein the crystal structure of the title compound

In the title molecule (Fig. 1), the benzofuran unit is essentially planar, with a mean deviation of 0.005 (1) Å from the least-squares plane defined by the nine constituent atoms. The carboxyl groups are involved in intermolecular O—H···O hydrogen bonds (Table 1), which link the molecules into centrosymmetric dimers.

### Experimental

Ethyl 2-(5-fluoro-7-methyl-3-methylsulfanyl-1-benzofuran-2-yl)acetate (338 mg, 1.2 mmol) was added to a solution of potassium hydroxide (337 mg, 6 mmol) in water (10 ml) and methanol (10 ml), and the mixture was refluxed for 5 h, then cooled. Water was added, and the solution was extracted with dichloromethane. The aqueous layer was acidified to pH 1 with concentrated hydrochloric acid and then extracted with chloroform, dried over magnesium sulfate, filtered and concentrated at reduced pressure. The residue was purified by column chromatography (ethyl acetate) to afford the title compound as a colorless solid [yield 82%, m.p. 436–437 K; *R*_f_ = 0.65 (ethyl acetate)]. Single crystals suitable for X-ray diffraction were prepared by evaporation of a solution of the title compound in benzene at room temperature.

### Refinement

All  H atoms were  positioned geometrically and refined using a riding model, with O—H = 0.84 Å, and C—H = 0.95 Å for aryl, 0.99 Å for methylene and 0.98 Å for methyl H atoms, respectively. *U*_iso_(H)  = 1.5*U*_eq_(O), and 1.2*U*_eq_(C) for aryl and methylene, and 1.5*U*_eq_(C) for methyl H atoms.

### Crystal data

**Table:**

C_12_H_11_FO_3_S	*F*(000) = 528
*M**_r_* = 254.27	*D*_x_ = 1.467 Mg m^−^^3^
Monoclinic, *P*2_1_/*c*	Mo *K*α radiation, λ = 0.71073 Å
Hall symbol: -P 2ybc	Cell parameters from 3638 reflections
*a* = 16.1747 (9) Å	θ = 2.5–27.9°
*b* = 4.9242 (3) Å	µ = 0.29 mm^−^^1^
*c* = 14.4572 (7) Å	*T* = 173 K
β = 91.701 (3)°	Block, colourless
*V* = 1150.97 (11) Å^3^	0.42 × 0.19 × 0.09 mm
*Z* = 4	

### Data collection

**Table:**

Bruker SMART APEXII CCD diffractometer	2873 independent reflections
Radiation source: rotating anode	2114 reflections with *I* > 2σ(*I*)
graphite multilayer	*R*_int_ = 0.050
Detector resolution: 10.0 pixels mm^-1^	θ_max_ = 28.4°, θ_min_ = 2.5°
φ and ω scans	*h* = −19→21
Absorption correction: multi-scan (*SADABS*; Bruker, 2009)	*k* = −6→6
*T*_min_ = 0.889, *T*_max_ = 0.975	*l* = −19→19
10241 measured reflections	

### Refinement

**Table:**

Refinement on *F*^2^	Primary atom site location: structure-invariant direct methods
Least-squares matrix: full	Secondary atom site location: difference Fourier map
*R*[*F*^2^ > 2σ(*F*^2^)] = 0.048	Hydrogen site location: difference Fourier map
*wR*(*F*^2^) = 0.153	H-atom parameters constrained
*S* = 1.05	*w* = 1/[σ^2^(*F*_o_^2^) + (0.0891*P*)^2^ + 0.0162*P*] where *P* = (*F*_o_^2^ + 2*F*_c_^2^)/3
2873 reflections	(Δ/σ)_max_ < 0.001
156 parameters	Δρ_max_ = 0.38 e Å^−^^3^
0 restraints	Δρ_min_ = −0.27 e Å^−^^3^

### Special details

**Table:**

Geometry. All esds (except the esd in the dihedral angle between two l.s. planes) are estimated using the full covariance matrix. The cell esds are taken into account individually in the estimation of esds in distances, angles and torsion angles; correlations between esds in cell parameters are only used when they are defined by crystal symmetry. An approximate (isotropic) treatment of cell esds is used for estimating esds involving l.s. planes.
Refinement. Refinement of F^2^ against ALL reflections. The weighted R-factor wR and goodness of fit S are based on F^2^, conventional R-factors R are based on F, with F set to zero for negative F^2^. The threshold expression of F^2^ > 2sigma(F^2^) is used only for calculating R-factors(gt) etc. and is not relevant to the choice of reflections for refinement. R-factors based on F^2^ are statistically about twice as large as those based on F, and R- factors based on ALL data will be even larger.

### Fractional atomic coordinates and isotropic or equivalent isotropic displacement parameters (Å^2^)

**Table:**

	*x*	*y*	*z*	*U*_iso_*/*U*_eq_	
S1	0.15190 (3)	0.70323 (12)	0.30067 (3)	0.0375 (2)	
F1	0.38947 (9)	−0.1018 (3)	0.42507 (7)	0.0606 (5)	
O1	0.29563 (7)	0.4092 (3)	0.10941 (7)	0.0292 (3)	
O2	0.10398 (10)	0.3674 (3)	0.03782 (12)	0.0558 (5)	
H2	0.0565	0.3189	0.0195	0.084*	
O3	0.05132 (8)	0.7769 (3)	0.01681 (10)	0.0438 (4)	
C1	0.22446 (10)	0.5379 (4)	0.23383 (11)	0.0273 (4)	
C2	0.28406 (10)	0.3388 (4)	0.26357 (10)	0.0255 (4)	
C3	0.30513 (12)	0.2144 (4)	0.34788 (11)	0.0324 (5)	
H3	0.2782	0.2579	0.4035	0.039*	
C4	0.36662 (11)	0.0275 (5)	0.34518 (11)	0.0355 (5)	
C5	0.40838 (11)	−0.0469 (4)	0.26625 (11)	0.0337 (5)	
H5	0.4506	−0.1811	0.2700	0.040*	
C6	0.38838 (10)	0.0749 (4)	0.18228 (11)	0.0291 (4)	
C7	0.32623 (11)	0.2652 (4)	0.18496 (10)	0.0257 (4)	
C8	0.23433 (10)	0.5719 (4)	0.14195 (11)	0.0286 (4)	
C9	0.43079 (12)	0.0035 (5)	0.09414 (12)	0.0402 (5)	
H9A	0.3896	−0.0592	0.0479	0.060*	
H9B	0.4713	−0.1410	0.1065	0.060*	
H9C	0.4591	0.1642	0.0707	0.060*	
C10	0.18996 (12)	0.7432 (5)	0.07219 (13)	0.0358 (5)	
H10A	0.2252	0.7646	0.0179	0.043*	
H10B	0.1815	0.9259	0.0989	0.043*	
C11	0.10771 (11)	0.6315 (4)	0.04024 (11)	0.0292 (4)	
C12	0.07261 (13)	0.4483 (5)	0.30180 (15)	0.0471 (6)	
H12A	0.0508	0.4187	0.2386	0.071*	
H12B	0.0278	0.5087	0.3411	0.071*	
H12C	0.0959	0.2782	0.3263	0.071*	

### Atomic displacement parameters (Å^2^)

**Table:**

	*U*^11^	*U*^22^	*U*^33^	*U*^12^	*U*^13^	*U*^23^
S1	0.0334 (3)	0.0289 (4)	0.0506 (3)	0.0038 (2)	0.0054 (2)	−0.0077 (2)
F1	0.0761 (9)	0.0696 (12)	0.0360 (6)	0.0303 (8)	−0.0012 (5)	0.0216 (6)
O1	0.0293 (6)	0.0322 (8)	0.0258 (5)	0.0010 (6)	−0.0025 (4)	0.0047 (5)
O2	0.0470 (9)	0.0267 (9)	0.0917 (11)	−0.0022 (8)	−0.0345 (8)	0.0036 (9)
O3	0.0319 (8)	0.0305 (9)	0.0680 (9)	0.0070 (7)	−0.0143 (7)	0.0012 (7)
C1	0.0244 (8)	0.0239 (10)	0.0335 (8)	−0.0010 (8)	−0.0007 (6)	−0.0001 (8)
C2	0.0238 (8)	0.0249 (10)	0.0279 (7)	−0.0015 (8)	0.0001 (6)	−0.0010 (7)
C3	0.0373 (10)	0.0343 (12)	0.0258 (7)	0.0002 (9)	0.0020 (7)	0.0027 (8)
C4	0.0401 (10)	0.0375 (13)	0.0286 (8)	0.0040 (10)	−0.0042 (7)	0.0097 (8)
C5	0.0296 (9)	0.0306 (12)	0.0406 (9)	0.0063 (9)	−0.0022 (7)	0.0001 (9)
C6	0.0246 (8)	0.0307 (12)	0.0321 (8)	−0.0019 (8)	0.0007 (6)	−0.0042 (8)
C7	0.0247 (8)	0.0278 (11)	0.0243 (7)	−0.0033 (8)	−0.0032 (6)	0.0021 (7)
C8	0.0240 (8)	0.0251 (11)	0.0362 (8)	−0.0015 (8)	−0.0052 (6)	0.0024 (8)
C9	0.0342 (10)	0.0504 (15)	0.0362 (9)	0.0065 (10)	0.0058 (7)	−0.0090 (9)
C10	0.0359 (10)	0.0290 (11)	0.0420 (9)	−0.0021 (9)	−0.0099 (8)	0.0080 (9)
C11	0.0324 (9)	0.0257 (11)	0.0291 (7)	−0.0007 (9)	−0.0042 (6)	0.0034 (8)
C12	0.0375 (11)	0.0407 (15)	0.0637 (12)	−0.0032 (11)	0.0121 (9)	−0.0064 (11)

### Geometric parameters (Å, °)

**Table:**

S1—C1	1.7440 (17)	C5—C6	1.384 (2)
S1—C12	1.795 (2)	C5—H5	0.9500
F1—C4	1.3605 (19)	C6—C7	1.376 (3)
O1—C8	1.369 (2)	C6—C9	1.507 (2)
O1—C7	1.382 (2)	C8—C10	1.484 (3)
O2—C11	1.303 (2)	C9—H9A	0.9800
O2—H2	0.8400	C9—H9B	0.9800
O3—C11	1.200 (2)	C9—H9C	0.9800
C1—C8	1.353 (2)	C10—C11	1.500 (3)
C1—C2	1.432 (3)	C10—H10A	0.9900
C2—C7	1.391 (2)	C10—H10B	0.9900
C2—C3	1.397 (2)	C12—H12A	0.9800
C3—C4	1.356 (3)	C12—H12B	0.9800
C3—H3	0.9500	C12—H12C	0.9800
C4—C5	1.392 (2)		
			
C1—S1—C12	99.86 (10)	C1—C8—O1	111.87 (15)
C8—O1—C7	105.96 (12)	C1—C8—C10	131.99 (18)
C11—O2—H2	109.5	O1—C8—C10	116.10 (15)
C8—C1—C2	106.36 (15)	C6—C9—H9A	109.5
C8—C1—S1	125.95 (15)	C6—C9—H9B	109.5
C2—C1—S1	127.69 (12)	H9A—C9—H9B	109.5
C7—C2—C3	119.05 (17)	C6—C9—H9C	109.5
C7—C2—C1	105.98 (14)	H9A—C9—H9C	109.5
C3—C2—C1	134.97 (16)	H9B—C9—H9C	109.5
C4—C3—C2	115.62 (15)	C8—C10—C11	114.05 (16)
C4—C3—H3	122.2	C8—C10—H10A	108.7
C2—C3—H3	122.2	C11—C10—H10A	108.7
C3—C4—F1	118.29 (15)	C8—C10—H10B	108.7
C3—C4—C5	125.18 (16)	C11—C10—H10B	108.7
F1—C4—C5	116.52 (18)	H10A—C10—H10B	107.6
C6—C5—C4	119.90 (18)	O3—C11—O2	123.63 (18)
C6—C5—H5	120.0	O3—C11—C10	121.85 (19)
C4—C5—H5	120.0	O2—C11—C10	114.49 (17)
C7—C6—C5	115.00 (15)	S1—C12—H12A	109.5
C7—C6—C9	122.29 (16)	S1—C12—H12B	109.5
C5—C6—C9	122.71 (17)	H12A—C12—H12B	109.5
C6—C7—O1	124.91 (14)	S1—C12—H12C	109.5
C6—C7—C2	125.25 (16)	H12A—C12—H12C	109.5
O1—C7—C2	109.83 (16)	H12B—C12—H12C	109.5
			
C12—S1—C1—C8	97.22 (19)	C9—C6—C7—C2	179.39 (18)
C12—S1—C1—C2	−81.79 (19)	C8—O1—C7—C6	179.17 (18)
C8—C1—C2—C7	−0.3 (2)	C8—O1—C7—C2	−0.02 (19)
S1—C1—C2—C7	178.89 (14)	C3—C2—C7—C6	0.3 (3)
C8—C1—C2—C3	−179.4 (2)	C1—C2—C7—C6	−179.00 (18)
S1—C1—C2—C3	−0.2 (3)	C3—C2—C7—O1	179.47 (16)
C7—C2—C3—C4	0.0 (3)	C1—C2—C7—O1	0.2 (2)
C1—C2—C3—C4	179.0 (2)	C2—C1—C8—O1	0.3 (2)
C2—C3—C4—F1	−179.57 (17)	S1—C1—C8—O1	−178.91 (13)
C2—C3—C4—C5	−0.3 (3)	C2—C1—C8—C10	178.18 (19)
C3—C4—C5—C6	0.5 (3)	S1—C1—C8—C10	−1.0 (3)
F1—C4—C5—C6	179.75 (17)	C7—O1—C8—C1	−0.2 (2)
C4—C5—C6—C7	−0.3 (3)	C7—O1—C8—C10	−178.42 (15)
C4—C5—C6—C9	−179.8 (2)	C1—C8—C10—C11	−77.6 (3)
C5—C6—C7—O1	−179.19 (16)	O1—C8—C10—C11	100.2 (2)
C9—C6—C7—O1	0.3 (3)	C8—C10—C11—O3	148.89 (18)
C5—C6—C7—C2	−0.1 (3)	C8—C10—C11—O2	−32.9 (2)

### Hydrogen-bond geometry (Å, °)

**Table:**

*D*—H···*A*	*D*—H	H···*A*	*D*···*A*	*D*—H···*A*
O2—H2···O3^i^	0.84	1.87	2.706 (2)	177

Symmetry codes: (i) −*x*, −*y*+1, −*z*.
